# Virus-like Silica Nanoparticles Improve Permeability of Macromolecules across the Blood–Brain Barrier In Vitro

**DOI:** 10.3390/pharmaceutics15092239

**Published:** 2023-08-30

**Authors:** Yuran Feng, Yuxue Cao, Zhi Qu, Taskeen Iqbal Janjua, Amirali Popat

**Affiliations:** School of Pharmacy, The University of Queensland, Brisbane, QLD 4102, Australia; yuran.feng@uq.net.au (Y.F.); yuxue.cao@uq.edu.au (Y.C.); zhi.qu@uqconnect.edu.au (Z.Q.)

**Keywords:** virus-like silica nanoparticles, blood–brain barrier, brain drug delivery, glioblastoma, surface roughness

## Abstract

The presence of the blood–brain barrier (BBB) limits the delivery of therapies into the brain. There has been significant interest in overcoming the BBB for the effective delivery of therapies to the brain. Inorganic nanomaterials, especially silica nanoparticles with varying surface chemistry and surface topology, have been recently used as permeation enhancers for oral protein delivery. In this context, nanoparticles with varying sizes and surface chemistries have been employed to overcome this barrier; however, there is no report examining the effect of nanoscale roughness on BBB permeability. This paper reports the influence of nanoscale surface roughness on the integrity and permeability of the BBB in vitro, using smooth surface Stöber silica nanoparticles (60 nm) compared to rough surface virus-like silica nanoparticles (VSNP, 60 nm). Our findings reveal that VSNP (1 mg/mL) with virus-mimicking-topology spiky surface have a greater effect on transiently opening endothelial tight junctions of the BBB than the same dose of Stöber silica nanoparticles (1 mg/mL) by increasing the FITC-Dextran (70 kDa) permeability 1.9-fold and by decreasing the trans-endothelial electrical resistance (TEER) by 2.7-fold. This proof-of-concept research paves the way for future studies to develop next-generation tailored surface-modified silica nanoparticles, enabling safe and efficient macromolecule transport across the BBB.

## 1. Introduction

Drug delivery to the brain is extremely challenging due to the presence of the blood–brain barrier (BBB). The BBB is a sophisticated and complex barrier made up of astrocytes, endothelial cells, neurons, and microglia which control the entry of substances and protect the central nervous system (CNS) [1]. Unlike other physiological barriers, the unique structure of the BBB, compromising tight junctions and the absence of any fenestrations, limits the free movement of the majority of substances [2]. The presence of tight junctions is a hallmark feature of the BBB. Tight junctions are highly specialised intercellular connections between adjacent endothelial cells that form the BBB to regulate the selective passage of substances into and out of the brain to maintain its homeostasis. The integrity of the BBB is necessary in order to prevent any harmful substances from crossing the brain; however, this presents a major challenge when it comes to delivering therapies for brain diseases [2,3,4]. For instance, while advances in cancer therapies have successfully been able to induce remission for breast or lung cancer, brain cancers still have poor prognosis, owing to the poor permeability of chemotherapies to the brain. Moreover, research suggests that the presence of an intact BBB plays a significant role in the treatment failure of various brain cancers such as Diffuse Intrinsic Pontine Glioma (DIPG) [5]. In fact, DIPG cells can directly impact vasculature to make the BBB and brain tumour barrier (BTB) less permeable, thereby reducing the effectiveness of therapies [5]. As such, there is a dire need to develop drug delivery platforms that can improve penetration across the BTB and BBB [6,7].

Nanotechnology-mediated drug delivery has made many advances in the last few decades. Numerous nanomedicines are already being used clinically and discussed in detail elsewhere [8,9,10]. There are significant advantages that nanomedicine offers which facilitate permeation across the BBB. Firstly, the small size of nanoparticles eases the passive transport across the BBB. Secondly, the surface of nanoparticles can be functionalized for active transport across the BBB via a receptor-mediated pathway to deliver drugs inside the brain. However, to date, no nanoparticle-based formulation to treat CNS diseases has been approved for clinical use because of poor efficacy [2]. Developing safe and effective nanoformulations for CNS applications requires meticulous consideration of various factors including the physicochemical properties of nanoparticles, such as size, surface chemistry, and stability. Given the challenges associated with treating CNS diseases and the lack of FDA-approved nanoformulations for the CNS, there is a pressing need for innovative approaches and the exploration of new strategies. By elucidating the factors contributing to the limited success thus far, we can identify potential avenues for innovation and pave the way for the development of safe and effective nanoformulations for the CNS.

Silica nanoparticles have gained significant prominence in recent years due to their applications in the field of targeted drug delivery and diagnostics, as evidenced by testing in numerous clinical trials [8]. Silica nanoparticles offer a key advantage due to their relative ease in modulating their physiochemical properties including surface area, shapes, size, and morphology [8,11,12,13,14]. The recent discovery of silica nanoparticles as novel permeation enhancers opened many avenues for their use in overcoming biological barriers. Silica nanoparticles open tight junctions in the gut by binding to the integrins on the cell surface, stimulating a cascade of signalling pathways and consequently triggering their rearrangement [15]. This effect is reported to be transient in nature, with particles showing no sign of toxicity in their assays. A recent paper published by Cao et al. found that silica nanoparticles with a rough surface and virus-like morphology further improved this effect and enabled the oral delivery of protein [16]. Surface roughness refers to the degree of variation or irregularity on the surface of the nanoparticle, which can be assessed using techniques such as transmission electron microscopy [17]. We hypothesised that the surface roughness of nanoparticles could possibly impact their interaction with the BBB in multiple ways [18,19]. Firstly, it can influence the adhesion and binding of the nanoparticles to the cells of the BBB [18]. Secondly, nanoparticles with a rough surface may exhibit a greater surface area, resulting in more interactions with proteins and other biomolecules in the bloodstream that could potentially interfere with their BBB transport [20]. Overall, the surface roughness of nanoparticles is an important parameter that can impact their interaction with the BBB and ultimately affect their ability to deliver therapeutic agents to the brain. Therefore, optimising the surface properties of nanoparticles, including their roughness, is a crucial step in developing effective nanomedicines for brain-related diseases. Based on the above literature, we hypothesise that the surface roughness could enhance nanoparticle interaction with the endothelial cells and transiently open tight junctions in the BBB, leading to controlled trafficking of substances across the BBB [16]. In this paper, we have evaluated the impact of virus-like silica on the transport of 70 kDa Dextran as a macromolecule model across the BBB in vitro.

## 2. Materials and Methods

### 2.1. Materials

Cetyltrimethylammonium bromide (CTAB), Tetraethoxysilane (TEOS), ammonium hydroxide, 3-Aminopropyltriethoxysilane (APTES), FITC-Carboxymethyl dextran 70 kD, and fetal bovine serum (FBS) were purchased from Sigma-Aldrich (Melbourne, Australia). Dimethyl sulfoxide (DMSO) was bought from Chem-supply (Adelaide, Australia). Cyanine 5 NHS ester (Cy5) dye was purchased from Lumiprobe Limited (Cockeysville, MD, USA, Asia & Pacific). Dulbecco’s Modified Eagle Medium (DMEM), penicillin/streptomycin (P/S), and 0.25% trypsin-EDTA were bought from Life Technologies (Brisbane, Australia). (4-(2-hydroxyethyl)-1-piperazineethanesulfonic acid) (HEPES) buffer was obtained from Gibco Australia. Pure ethanol was purchased from the University of Queensland Science store.

The human brain endothelial cell line (hCMEC/D3) (lot#SCC066) was obtained from Millipore Merck (Melbourne, Australia). Alamar Blue Assay Kit and cell staining dye were bought from Thermofisher (Brisbane, Australia) and included: DAPI (4′,6-diamidino-2-phenylindole), Alexa Fluor 594 conjugated anti-Zonula occludens-1 (ZO-1) antibodies, and Alexa Fluor 488 conjugated anti-Claudin 5 antibodies.

### 2.2. Synthesis of Silica Nanoparticles

#### 2.2.1. Virus-like Silica Nanoparticles (VSNP)

VSNP were synthesised using a previously optimised method [16]. Briefly, CTAB (1000 mg) was mixed in water (50 mL) and NaOH (0.1 M, 0.8 mL) using a round bottom flask. The mixture was heated to 60 °C and stirred at 1000 rpm. After 30 min, cyclohexane (20 mL) with tetraethoxysilane (TEOS, 98%) (20 *v*/*v* %) was then added dropwise into the round bottom flask and stirred for 48 h at 1000 rpm. Pellets were collected by centrifugation at 24,700× *g* for 10 min and washed with water and ethanol three times. The obtained particles were calcined using a muffle furnace (Thermo Scientific, Melbourne, Australia) at 550 °C for 5 h to remove any excess surfactants. CTAB was added as the structure-directing agent which aids in shaping the spiky structure on the surface of silica nanoparticles [21]. CTAB molecules self-assemble in an aqueous solution to form micelles due to their amphiphilic nature. These micelles serve as scaffolds for the growth of VSNP. CTAB’s presence directs the growth of “necks” on the silica nanoparticles to provide a virus-like morphology. As CTAB is an organic compound and exposure to high temperatures of 550 °C for 5 h causes it to decompose, which could remove CTAB molecules from the VSNP.

#### 2.2.2. Stöber Silica Nanoparticles

Stöber nanoparticles were synthesised using a previously optimised method [16]. Briefly, a mixture containing water (6.8 mL), ethanol (17.6 mL), and ammonium hydroxide (0.7 mL) was prepared in a 100 mL Schott bottle. Then, a premixed TEOS (1.4 mL) in ethanol (22.2 mL) was added dropwise to the Schott bottle. The mixture was stirred for 13 h at 500 rpm and the temperature was maintained at 60 °C. The Stöber nanoparticles were collected by centrifugation at 24,700× *g* for 10 min and subsequently washed thrice with water and ethanol. The obtained particles were calcined at 550 °C for 5 h.

### 2.3. Fluorescent Dye Cy5 Labelling on Silica Nanoparticles

First, the VSNP and Stöber nanoparticles were amine functionalised by adding corresponding nanoparticles (100 mg) in 30 mL water-free toluene and then refluxed at 115 °C using our previously established methods [16,22,23,24,25]. After 1 h, 100 µL 3-aminopropyltriethoxysilane (APTES) was added to the mixture, followed by further refluxing for 18 h. Nanoparticles were collected by centrifugation at 24,700× *g* for 10 min and then washed with ethanol and water 3 times to remove access unreacted silane. The product was obtained by drying in the oven for 24 h at 60 °C.

Cy-5 labelling of silica nanoparticles was performed using previously established methods [16,23,24,26]. The Cy5 dye solution (0.6 mL, 1 mg/mL in DMSO) was added to 30 mg amino-functionalized nanoparticles (suspended in DMSO 10 mg/mL) and stirred for 2 h. The final product was collected by centrifugation (24,700× *g*, 5 min), washed five times with ethanol, and vacuum dried for 24 h at room temperature.

### 2.4. Physiochemical Characterisation of Nanoparticles

The prepared VSNP and Stöber nanoparticles were characterised by their physiochemical properties. The shape and size of the nanoparticles were assessed using the Transmission Electron Microscopy (TEM) HITACHI HT7700 machine at a voltage of 80 kV (Tokyo, Japan). A carbon-coated copper grid was used to prepare the samples and the nanoparticles (1 mg/mL sonicated for 10 min in ethanol) were added dropwise and air-dried prior to imaging. The Zeta potential of the nanoparticles was quantified using a Dynamic light scattering (DLS) Nano series ZS instrument (Malvern Panalytical, Birmingham, UK) by suspending nanoparticles in water (0.01 mg/mL), and the experiment was performed in triplicate.

The surface area of the nanoparticles was measured by nitrogen absorption–desorption at 77 K using a Micromeritics Tristar II 3020 system (Micrometrics Tristar II, United Kingdom), and was then calculated using the Brunauer–Emmett–Teller (BET) method. Around 60 mg of nanoparticle was degassed (60 °C) for 24 h using vacuum drying (300 mBar) and analysis was conducted under liquid nitrogen.

### 2.5. Cell Culture

Cells were maintained as per the previous methods [23,26]. For the in vitro BBB transwell model, the human brain endothelial hCMEC/D3 cell line was selected and grown with an EndoGRO-MV Complete Culture Media Kit enhanced with 1 ng/mL FGF-2. Cells were grown on collagen-coated tissue culture flasks (Gibco Collagen Type I Rat Tail (150 μg/mL) diluted in acetic acid (10 mL, 20 mM)). Cells were passaged using 0.25% trypsin−EDTA every 2–3 days. When they reached 80–90% confluence and were kept under conditions of 37 °C, 5% CO_2_, and 95% humidity. hCMEC/D3 endothelial cells with passage numbers between 7 and 10 were used for experiments.

### 2.6. Cell Viability

Human brain endothelial hCMEC/D3 cells were plated in Corning 96-well plates at a cell density of 10,000 cells per well and subsequently incubated for 24 h. Nanoparticles dispersed in media with different concentrations (10 µg/mL, 100 µg/mL, and 1000 µg/mL) were then added to the cells. After 4 h of incubation, the cells were rinsed with PBS to remove the particles. Fresh media mixed with 10% Alamar Blue was added to the cells and measured for fluorescence on a spectrophotometer (FLUOstar Omega, BMG Labtech, Melbourne, Australia) at an excitation wavelength of 544 nm and an emission wavelength of 590 nm after 1 h incubation with Alamar Blue. Results were analysed as per manufacturers’ guidelines using the following formula:

$$\frac{FI\;590\;of\;test\;agent\;dilution}{FI\;590\;of\;untreated\;control}\times 100$$

where FI 590 represents the fluorescent intensity at an emission wavelength of 590 nm.

### 2.7. Cellular Uptake

Human brain endothelial hCMEC/D3 cells were plated for 24 h on 12-well plates (Cellvis glass bottom). Then, Cy5-labelled VSNP and Stöber nanoparticles were, respectively, mixed in the cell culture media (10 µg/mL and 100 µg/mL) and the treatments were left for 24 h prior to analysis. The cells were first fixed using PFA (4%, 20 min), followed by washing with ice-cold PBS (4 °C) 3 times and the addition of Triton-X100 (0.2%, 5 min). Cell staining solution Alexa Fluor™ 488 Phalloidin was added (30 min) as per the manufacturer’s advice, followed by the addition of DAPI (3 µM, 5 min). Cellular uptake of Cy5-labelled nanoparticles onto hCMEC/D3 cells was observed using the laser scanning confocal microscope Olympus FV3000 at 40× magnification.

### 2.8. In Vitro Transwell BBB Model

**Figure 1:**
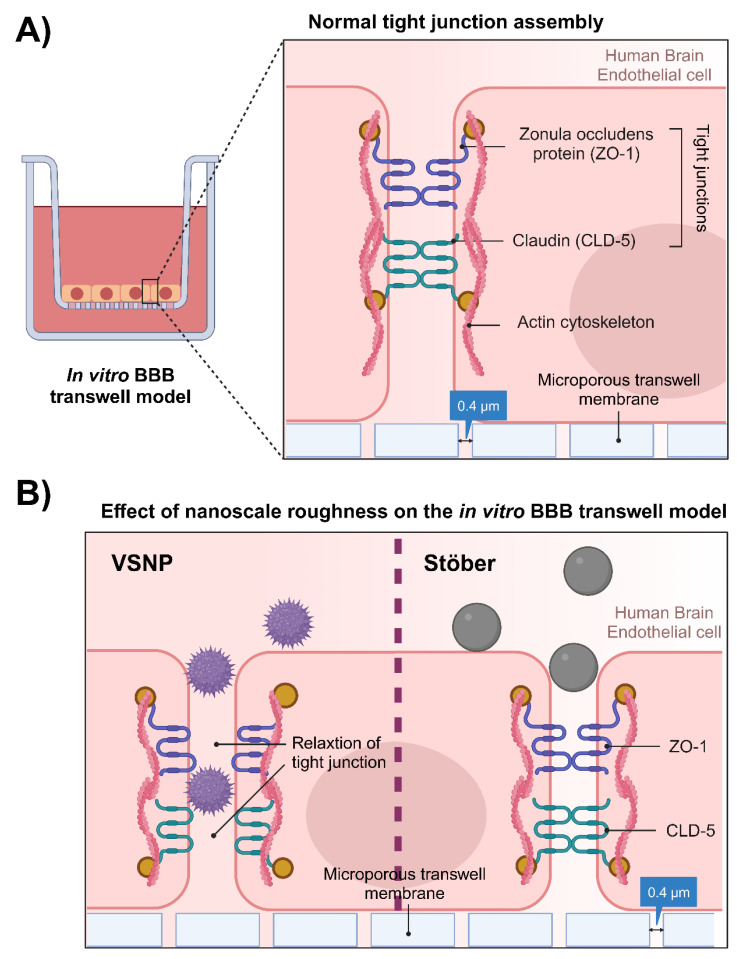
Establishment of the human brain endothelial in vitro transwell model utilizing hCMEC/D3 monolayers. (**A**) Schematic diagram of the hCMEC/D3 monolayers’ layout and the tight junctions that control paracellular permeability in the transwell system. (**B**) Illustration of the influence of nanoscale roughness on the BBB permeability by inducing the relaxation of tight junctions. Created with BioRender.com.

A previously developed in vitro BBB transwell model using human brain endothelial hCMEC/D3 cell lines was utilised, as shown in Figure 1A [26,27]. Briefly, the transwell plate (12 wells) was first collagen-coated (200 μL, 150 μg/mL) for 1 h. Cells were then plated in the apical chamber of the transwell (2.5 × 10^4^ cells/cm^2^) and grown for 7 days. Media in both the apical and basolateral sides was replaced every 2 days. Then, VSNP and Stöber nanoparticles were, respectively, mixed with cell culture media (concentration range 10 µg/mL to 1000 µg/mL) and added to the apical chamber of the in vitro transwell BBB model. The corresponding TEER values were recorded hourly. After 4 h of treatment, the apical chamber was rinsed with PBS to remove the nanoparticles and replaced with culture media to assess the tight junction recovery. The experiment was performed in triplicates. The effect of nanoparticles interacting with the tight junction in vitro transwell BBB was assessed (Figure 1B).

Trans-endothelial electrical resistance (TEER) and the phenotypic expression of tight junction zonula occludens-1 (ZO-1) and claudin-5 (CLD-5) present on the BBB monolayer were evaluated to ensure the integrity of the barrier was achieved. TEER was measured using an EVOM volt-ohmmeter (World Precision Instruments, Sarasota, FL, USA) and experiments were performed at ≥50 Ω.cm^2^. Separate in vitro BBB monolayers were prepared to measure TEER or tight junction staining using confocal microscopy.

The tight junction proteins ZO-1 and CLD-5 were stained and their expression was assessed using confocal microscopy to image the in vitro hCMEC/D3 monolayer as per previous methods [26]. Briefly, the in vitro BBB monolayer was washed thrice with ice-cold PBS (containing Ca^2+^ and Mg^2+^ ion 0.1 g/L) to ensure the cell remained attached during subsequent treatments and washing procedures. Then, cold methanol (4 °C) was used to fix the cell at −20 °C. After 20 min, the monolayer was washed three times with PBS, then TritonX-100 (0.1% in PBS) was added and washed after 5 min with PBS three times. Non-specific antibody binding was blocked by adding the BSA (0.2% in PBS) for 1 h at room temperature. Immunostaining of ZO-1 and CLD-5 (20 µg/mL) was then added and stored at 4 °C overnight. Unreacted stains were washed with PBS and DAPI (3 µM for 5 min) was added and washed with PBS before analysis under the laser scanning confocal microscope Olympus FV3000 at 40× magnification.

### 2.9. Modulation of In Vitro BBB Model for Transport of Macromolecules

We used 70 kDa FITC dextran molecules to assess whether the in vitro modulation of the BBB using rough nanoparticles can be utilised for the transport of macromolecules. First, VSNP and Stöber were added (1000 µg/mL) to transiently open the BBB tight junctions. After 1 h, 70 kDa FITC dextran was added (200 μg/mL) to the apical chamber of the transwell. The control group was not given nanoparticles or dextran. TEER was measured every hour and a media sample (100 μL) was collected from the basolateral chamber to measure the permeation of 70 kDa FITC dextran. Fluorescein isothiocyanate (FITC) is a derivative of fluorescein. Due to its remarkable quantum efficiency and stable conjugate, FITC is widely employed as a fluorescent labelling reagent in a wide range of applications [28,29]. The experiment was performed in triplicate. The fluorescence intensity values of dextran were measured (excitation wavelength: 493 nm; emission wavelength: 517 nm, FLUOstar Omega, BMG). Fluorescence intensity values were compared between the control (dextran alone) and treatment of VSNP with dextran or Stöber with dextran.

### 2.10. Statistical Analysis

GraphPad Prism 9.0 software was used for all statistical analyses (GraphPad Software, San Diego, CA, USA). Changes in fluorescence intensity in vitro in the BBB model were analysed by a two-way ANOVA method, whereas TEER values were analysed by one-way ANOVA.

## 3. Results and Discussion

### 3.1. Synthesis, Functionalization, and Characterization of Nanoparticles

**Figure 2:**
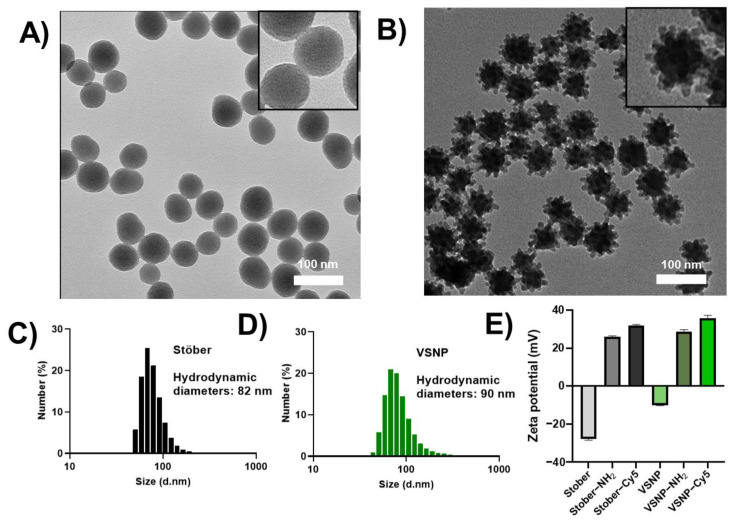
Representative transmission electron microscope images of (**A**) 60 nm Stöber silica nanoparticles and their enlarged image (inset) and (**B**) 60 nm virus-like silica nanoparticles (VSNP) and their enlarged image (inset). The scale bar is 100 nm. (**C**,**D**) Size distribution of Stöber and VSNP measured by dynamic light scattering (n = 3). (**E**) Zeta potential of the Stöber and VSNP (n = 3).

Stöber silica nanoparticles and 60 nm sized VSNP were synthesised as per previous reports [16]. Both nanoparticles showed a monodispersed and uniform particle size and shape, as evidenced by TEM images and DLS data (Figure 2A–C and Table 1). As expected, a larger size is measured by DLS when compared to TEM due to the presence of a hydrodynamic layer, and this phenomenon has been extensively documented and corroborated by numerous researchers in the field of nanomedicine [23,24,30]. The adsorption–desorption isotherms for nitrogen are shown in Figure S1. The surface area of Stöber was found to be 78 m^2^/g compared to 117 m^2^/g for VSNP (Table 1), which can be attributed to the rough morphology of VSNP, and this effect has been reported in the literature [17].

Next, amino functionalization was first performed on particles by the covalent attachment of APTES. Specifically, a post-synthesis grafting method was used under anhydrous conditions, employing organosilane surface modifier APTES molecules which react with hydroxyl groups found on the surface of silica nanoparticles. This was demonstrated by the change in the zeta potential of the particles from negative to positive (Figure 2E). The fluorescent dye Cy5 was then covalently attached to the amino group of nanoparticles via standard EDC/NHS-coupling chemistry. The amino-functionalized nanoparticles remained positively charged after the attachment of Cy5 (Figure 2E).

### 3.2. Cell Viability and Cellular Uptake

The cell viability of hCMEC/D3 was measured after treatment with different concentrations of Stöber and VSNP. The human brain endothelial cell hCMEC/D3 is a primary cell line isolated from human temporal lobe microvessels. It is important to use a dose of silica nanoparticles that does not induce cytotoxic effects. It was found that after treatment with either Stöber or VSNP in different concentrations (10, 100, and 1000 µg/mL), the hCMEC/D3 had above 80% cell viability, demonstrating a low cytotoxic potential of nanoparticles (Figure S2). Similar findings have been observed on other cell lines using these silica nanoparticles [16].

Next, investigations were carried out to determine if the differences in surface roughness had an impact on the cellular uptake of silica nanoparticles in the hCMEC/D3 cell line. Nanomaterials with topological structures mimicking viruses have garnered significant interest [31]. Certain viruses, such as the SARS-CoV-2 virus responsible for COVID-19, have spike proteins on their surface that have been shown to play a crucial role in cellular uptake [32]. These spike proteins lead to the characteristic virus-like-topology, facilitating the attachment of the virus to the surface of host cells, enabling cellular uptake, and ultimately initiating infection [21,33]. It has been reported that nanotopographic materials exert specific biophysical cues that modulate tight junctions and ultimately can influence their barrier function [15,16,31]. In this experiment, the hCMEC/D3 cells were exposed to varying concentrations of Stöber and VSNP to elucidate the influence of surface roughness on cellular uptake. Owing to their relatively small size, both Stöber and VSNP were taken up by the endothelial cells (Figure S3). However, it was found that VSNP had better interaction and cellular uptake when compared to Stöber nanoparticles (Figure S3). Even at low concentrations of 10 µg/mL, VSNP are taken by hCMEC/D3 cells, but this is not the case for Stöber (Figure S3). The differences in cellular uptake can be explained by differences in surface morphology; the mimicry of the virus-like structure of VSNP along with a comparatively higher surface area is more likely to have better cellular interaction [21].

### 3.3. Effect of Silica Nanoparticles on the Integrity of the Blood–Brain Barrier In Vitro

**Figure 3:**
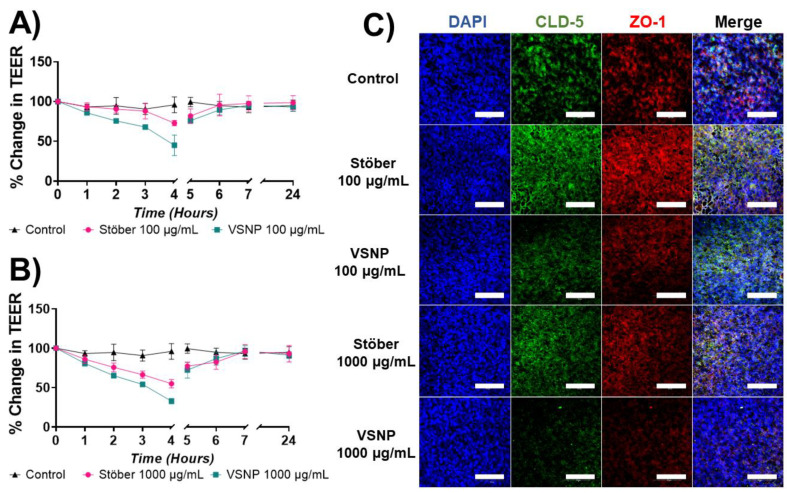
Influence of Stöber and VSNP on the integrity of the blood–brain barrier. Concentrations of 100 and 1000 µg/mL were added to the apical chamber of the in vitro BBB transwell model using hCMEC/D3 endothelial cells. Changes in the TEER value after the addition of nanoparticles at concentrations (**A**) 100 µg/mL, (**B**) 1000 µg/mL. After four hours the monolayers were washed, and recovery of TEER was measured. (**C**) Confocal images showing the influence of Stöber and VSNP on tight junctions of green Claudin-5 (CLD-5) and red Zonna-Occludin-1 (ZO-1) after four hours. Blue DAPI represent nuclei. The scale bar is 100 µm. Experiments were performed in triplicate. Scale bar: 100 µm.

**Figure 4:**
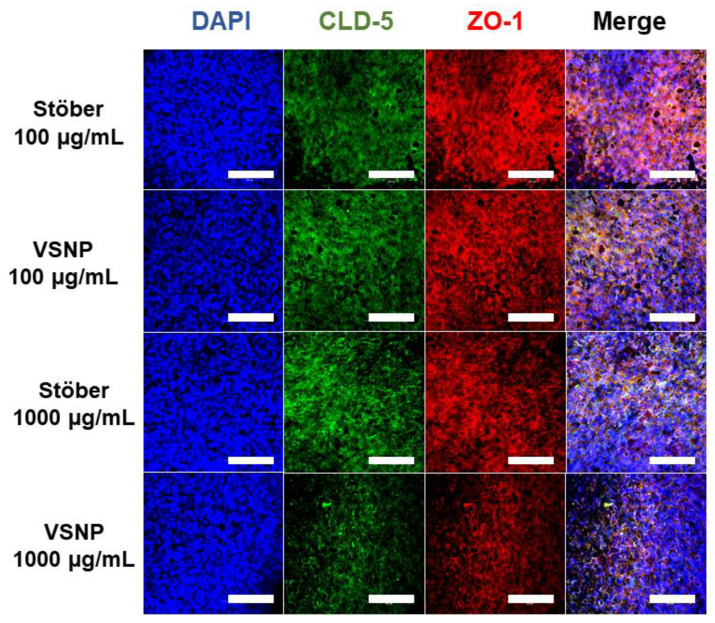
Recovery of tight junctions present on the in vitro transwell blood–brain barrier. After the removal of Stöber and VSNP, transwells were washed and placed in the incubator for 24 h. Recovery of the integrity of the BBB can be observed, as evidenced by the presence of tight junctions of green Claudin-5 (CLD-5) and red Zonna-Occludin-1 (ZO-1) post-treatment with Stöber and VSNP nanoparticles in the confocal images. Scale bar: 100 µm.

The integrity of BBB is controlled by the presence of tight junctions—a type of protein complexes involved in linking neighbouring endothelial cells to maintain a cellular strong adhesion. In this work, we investigated the influence of the surface roughness of silica nanoparticles on tight junctions, specifically the Zonula occludens-1 (ZO-1) and Claudin-5 (CLD-5) [34]. Both the ZO-1 and CLD-5 have been reported to play a crucial role in establishing and maintaining strong and tight contacts between adjoining endothelial cells [35]. To ensure the in vitro transwell BBB model was representative of the physiological conditions, the TEER was assessed as an indicator for strong BBB barrier integrity. After the addition of Stöber and VSNP, the TEER value dropped. VSNP had a greater drop in TEER when compared to Stöber at both concentrations (100 and 1000 µg/mL) (Figure 3A,B), which could be attributed to their increased cell adhesion and ability to have multiple contact points due to the spike shaped surface morphology [36]. The changes in TEER were further corroborated by the reduced expression of ZO-1 and CLD-5 via confocal microscopy. In the control group without nanoparticles, the presence of tight junctions between the cells was evident from the fluorescent signal of both CLD-5 (green) and ZO-1 (red). However, the fluorescent signal of CLD-5 (green) and ZO-1 (red) reduced after the treatment of VSNP and Stöber, indicating the potential disruption of tight junctions [15,16]. When compared to Stöber, the VSNP had a more pronounced effect on the tight junction disruption and less fluorescent signal was observed at each concentration for both tight junctions (CLD-5 and ZO-1) (Figure 3C). A reduction in fluorescent signal becomes noticeable as the concentration of silica nanoparticles is increased. This phenomenon was particularly pronounced in the 1000 µg/mL dose group, wherein the ZO-1 and CLD-5 staining was barely visible in the VSNP-treated group. The absence of staining for both ZO-1 and CLD-5 suggested a disruption of tight junctions, resulting in weakened intercellular interactions and a reduction in TEER measurements. More importantly, the recovery of the tight junction can be observed from the TEER values and confocal images of all the treatment groups after twenty-four hours (Figure 4), demonstrating that the influence of nanoparticles in opening the tight junction is transient and reversible. The differences in surface morphology, specifically the higher surface area of VSNP compared to Stöber, enabled a higher degree of interaction with the in vitro BBB monolayer, which then led to transient relaxation of tight junctions. In addition to the higher surface area of VSNP, the higher degree interaction of VSNP with the BBB monolayer can be attributed to the virus mimicking irregular surfaces and the nanotopographics of VSNP, causing an increase in surface bio-adhesion to the BBB monolayer [36]. This was evident by two different methods of analysis, i.e., physical changes to the electrical resistance of the monolayer as measured by TEER and loss of the fluorescent signal of ZO-1 and CLD-5, as measured by confocal imaging. Altogether, the in vitro transwell BBB model demonstrated that the VSNP exhibited superior ability compared to Stöber nanoparticles to temporarily disrupt the tight junctions.

### 3.4. Transient Relaxation of Tight Junctions in the Blood–Brain Barrier for Macromolecule Delivery

Nanoparticles with sizes smaller than 40 nm have been reported to exhibit better permeability on the BBB [37,38,39], but there are no studies on the effect of surface roughness of nanoparticles across the BBB. Therefore, we next investigated if the surface roughness of small silica nanoparticles could enhance the transport of macromolecule 70 kDa Dextran across the in vitro BBB model.

**Figure 5:**
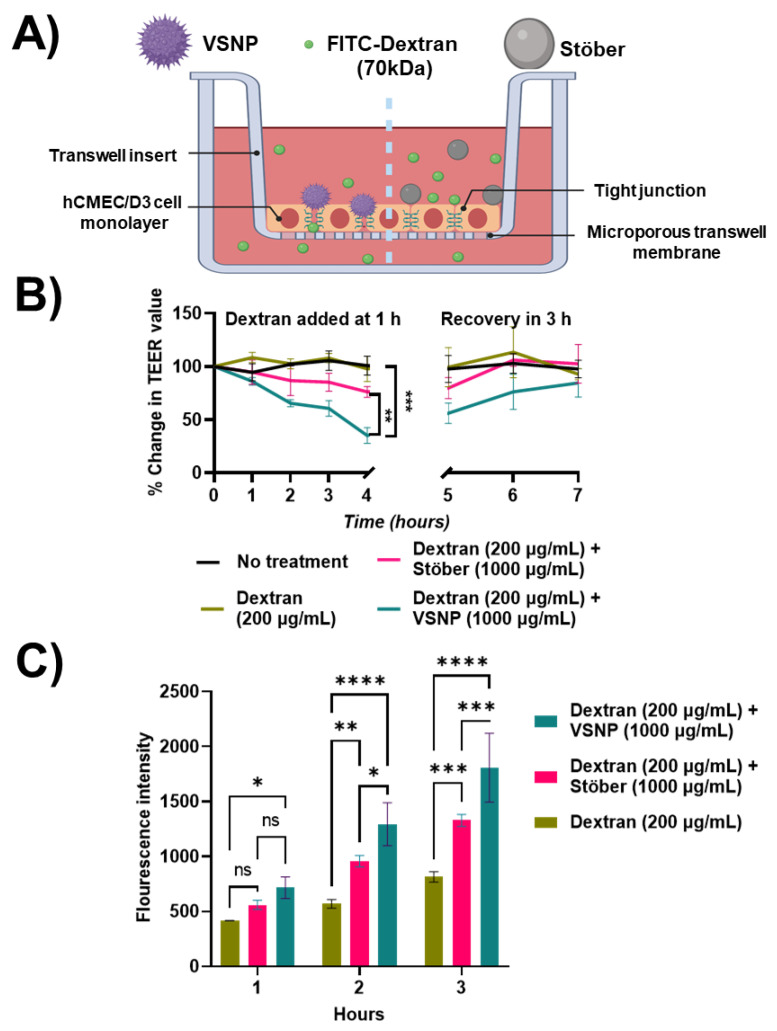
Influence of Stöber and VSNP on the transport of macromolecules across the in vitro transwell blood–brain barrier. (**A**) Schematic showing the layout of the in vitro transwell system; Stöber and VSNP were added into the apical chamber with 70 kDa FITC Dextran, respectively, (**B**) TEER values measured as a function of time after exposure to 1000 µg/mL of either Stöber or VSNP nanoparticles, n = 3. (**C**) The fluorescence intensity of FITC Dextran 70 kDa in the basal chamber at different time points. ns: *p* > 0.05, * *p* < 0.05, ** *p* < 0.01, *** *p* < 0.001, **** *p* < 0.0001, n = 3. Changes in fluorescence intensity in vitro BBB model were analysed by a two-way ANOVA method whereas TEER values were analysed by one-way ANOVA.

As a large hydrophilic macromolecule with hydrodynamic radius of 6 nm, 70 kDa Dextran does not usually permeate the BBB (reported P_app_ from literature ~1 × 10^–6^ cm/s) [26,27]. From the literature, it has been reported that smaller molecules such as 4 kDa dextran typically have better permeabilities (P_app_ ~5–13 × 10^–6^ cm/s) [27]. Therefore, 70 kDa FITC labelled Dextran was selected as a model substance for macromolecule transport across the BBB. First, the VSNP and Stöber nanoparticles (1000 µg/mL) were added to the apical chamber of the in vitro BBB monolayer (Figure 5A). After 1 h, 70 kDa FITC labelled Dextran was added to the apical chamber and its permeation across the in vitro BBB monolayer was assessed. As anticipated, the TEER values dropped for both Stöber (35%) and VSNP (76%), and the VSNP induced a significantly greater drop in the TEER value (Figure 5B). Additionally, the amount of 70 kDa Dextran that permeated in the basal chamber was simultaneously examined at each time point as a function of its fluorescence intensity (Figure 5C). VSNP treated BBB transwells showed a significant FITC fluorescence signal coming from the 70 kDa dextran molecules when compared to control and Stöber nanoparticles at 2 and 3 h (Figure 5C) but not at 1 h. Our data demonstrated that compared to a smooth surface the presence of a rough surface can enhance the in vitro BBB permeability of macromolecules such as 70 kDa dextran by promoting increased interaction and subsequently inducing relaxation of tight junctions. This phenomenon was not seen in the control groups, demonstrating the crucial interplay of the surface roughness in manipulating the tight junction relaxation. Overall, it was found that when compared to Stöber, the VSNP had a superior ability to transiently induce the relaxation of tight junctions, as demonstrated by TEER changes, and thereby improve 70 kDa FITC dextran permeation across the BBB using the in vitro transwell BBB model.

## 4. Conclusions

In conclusion, the surface roughness of silica nanoparticles plays a crucial role in their interaction with the tight junction produced by brain endothelial cells (hCMEC/D3) in vitro. In this work we report for the first time that the surface roughness of nanoparticles can transiently affect the BBB permeability to enable the transport of macromolecules across the BBB. When compared to the smooth surface of Stöber silica nanoparticles, the rough surfaces of virus-like silica nanoparticles (VSNP) tend to have a greater effect on transiently opening tight junctions present on the BBB to allow transport of macromolecules such as 70 kDa Dextran. The influence of surface roughness of silica nanoparticles on the BBB integrity is attributed to the altered cellular interactions with tight junctions, which can be measured directly by changes to trans-endothelial resistance (TEER) and difference in tight junction phenotypic expression as assessed by confocal microscopy. Moreover, the findings suggest that the effect of rough surface silica nanoparticles is transient and TEER was recovered within 8 h. While our investigation did not compare anionic and cationic nanoparticles directly, rough surface VSNP anionic nanoparticles have demonstrated notable permeation enhancing ability, highlighting the significance of the anionic characteristics in facilitating the transport of macromolecules. These findings can potentially be utilised in the future to improve the drug delivery of large molecules which currently are unable to permeate the BBB and have the potential to improve therapies for neurodegenerative diseases and brain-targeted therapies. Nevertheless, further studies are required using more complex 3D and preclinical animal models to comprehensively understand the underlying mechanisms and to develop tailored surface modifications that promote safe and efficient macromolecule transport across the BBB. Overall, the investigation into the influence of silica nanoparticle surface roughness on the BBB represents a significant step towards advancing nanomedicine and its applications in the field of brain drug delivery.

## Figures and Tables

**Table 1:** Physical characterisation of silica nanoparticles, Stöber and VSNP. The average size was calculated by measuring the diameter of 100 nanoparticles using TEM images and analysed by Image J software (version 1.53t). The surface area of the nanoparticles and the zeta potential as measured by Brunauer–Emmett–Teller (BET) and DLS instruments, respectively.

Sample	Average Size nm (Measured via TEM)	BET Surface Area (m^2^g^−1^)
Stöber	59 ± 6	78
VSNP	62 ± 10	117

## Data Availability

Data is contained within the article or supplementary material.

## Supplementary Materials

The following supporting information can be downloaded at: https://www.mdpi.com/article/10.3390/pharmaceutics15092239/s1, Figure S1: Nitrogen adsorption−desorption isotherms of (A) Stöber and (B) VSNP; Figure S2: Cell viability was test in hCMEC/D3 brain endothelial cell line. Cell treated with either Stöber and VSNP for 4 h (concentration from 10 µg/mL to 1000 µg/mL) showed no significant toxicity and cell viability was above 80% at all concentrations. AlamarBlue assay was performed to study the cytotoxicity, n = 5 for treatment groups and n = 10 for no treatment group; Figure S3: Uptake of Stöber and VSNP nanoparticles into human brain endothelial cells hCMEC/D3 analysed by confocal microscopy. Scale bar, 100 μm. Blue DAPI staining nuclei, green Phalloidin staining cytoskeleton and pink Cy5 labelled silica nanoparticles.

## References

[1] Kadry H., Noorani B., Cucullo L. (2020). A blood–brain barrier overview on structure, function, impairment, and biomarkers of integrity. Fluids Barriers CNS.

[2] Janjua T.I., Rewatkar P., Ahmed-Cox A., Saeed I., Mansfeld F.M., Kulshreshtha R., Kumeria T., Ziegler D.S., Kavallaris M., Mazzieri R. (2021). Frontiers in the treatment of glioblastoma: Past, present and emerging. Adv. Drug Deliv. Rev..

[3] Daneman R., Prat A. (2015). The blood–brain barrier. Cold Spring Harb. Perspect. Biol..

[4] Pardridge W.M. (2007). Blood–brain barrier delivery. Drug Discov. Today.

[5] Ung C., Upton D.H., Venkat P., Pandher R., Curnis F., Corti A., Nicolazzo J., Mayoh C., Tsoli M., Ziegler D.S. (2022). DIPG-21. DIPG cells alter the permeability of the blood-brain barrier in the brainstem leading to treatment failure. Neuro-Oncology.

[6] Thakur A., Faujdar C., Sharma R., Sharma S., Malik B., Nepali K., Liou J.P. (2022). Glioblastoma: Current Status, Emerging Targets, and Recent Advances. J. Med. Chem..

[7] Marenco-Hillembrand L., Wijesekera O., Suarez-Meade P., Mampre D., Jackson C., Peterson J., Trifiletti D., Hammack J., Ortiz K., Lesser E. (2020). Trends in glioblastoma: Outcomes over time and type of intervention: A systematic evidence based analysis. J. Neuro-Oncol..

[8] Janjua T.I., Cao Y., Yu C., Popat A. (2021). Clinical translation of silica nanoparticles. Nat. Rev. Mater..

[9] Anselmo A.C., Mitragotri S. (2021). Nanoparticles in the clinic: An update post COVID-19 vaccines. Bioeng. Transl. Med..

[10] Huang H., Feng W., Chen Y., Shi J. (2020). Inorganic nanoparticles in clinical trials and translations. Nano Today.

[11] Manzano M., Vallet-Regí M. (2020). Mesoporous silica nanoparticles for drug delivery. Adv. Funct. Mater..

[12] Vallet-Regí M., Schüth F., Lozano D., Colilla M., Manzano M. (2022). Engineering mesoporous silica nanoparticles for drug delivery: Where are we after two decades?. Chem. Soc. Rev..

[13] Schmid R., Neffgen N., Lindén M. (2023). Straightforward adsorption-based formulation of mesoporous silica nanoparticles for drug delivery applications. J. Colloid Interface Sci..

[14] Theivendran S., Lazarev S., Yu C. (2023). Mesoporous silica/organosilica nanoparticles for cancer immunotherapy. Exploration.

[15] Lamson N.G., Berger A., Fein K.C., Whitehead K.A. (2020). Anionic nanoparticles enable the oral delivery of proteins by enhancing intestinal permeability. Nat. Biomed. Eng..

[16] Cao Y., Janjua T.I., Qu Z., Draphoen B., Bai Y., Linden M., Moniruzzaman M., Hasnain S., Kumeria T., Popat A. (2023). Virus-like silica nanoparticles enhance macromolecule permeation in vivo. Biomater. Sci..

[17] Niu Y., Yu M., Meka A., Liu Y., Zhang J., Yang Y., Yu C. (2016). Understanding the contribution of surface roughness and hydrophobic modification of silica nanoparticles to enhanced therapeutic protein delivery. J. Mater. Chem. B.

[18] Wang P., Wang C., Lu L., Li X., Wang W., Zhao M., Hu L., El-Toni A.M., Li Q., Zhang F. (2017). Kinetics-mediate fabrication of multi-model bioimaging lanthanide nanoplates with controllable surface roughness for blood brain barrier transportation. Biomaterials.

[19] Wang X., Hou Y., Ai X., Sun J., Xu B., Meng X., Zhang Y., Zhang S. (2020). Potential applications of microfluidics based blood brain barrier (BBB)-on-chips for in vitro drug development. Biomed. Pharmacother..

[20] Guo P., Liu D., Subramanyam K., Wang B., Yang J., Huang J., Auguste D.T., Moses M.A. (2018). Nanoparticle elasticity directs tumor uptake. Nat. Commun..

[21] Wang W., Wang P., Tang X., Elzatahry A.A., Wang S., Al-Dahyan D., Zhao M., Yao C., Hung C.-T., Zhu X. (2017). Facile synthesis of uniform virus-like mesoporous silica nanoparticles for enhanced cellular internalization. ACS Cent. Sci..

[22] Meka A.K., Gopalakrishna A., Iriarte-Mesa C., Rewatkar P., Qu Z., Wu X., Cao Y., Prasadam I., Janjua T.I., Kleitz F. (2023). Influence of Pore Size and Surface Functionalization of Mesoporous Silica Nanoparticles on the Solubility and Antioxidant Activity of Confined Coenzyme Q10. Mol. Pharm..

[23] Janjua T.I., Ahmed-Cox A., Meka A.K., Mansfeld F.M., Forgham H., Ignacio R.M.C., Cao Y., McCarroll J.A., Mazzieri R., Kavallaris M. (2021). Facile synthesis of lactoferrin conjugated ultra small large pore silica nanoparticles for the treatment of glioblastoma. Nanoscale.

[24] Chaudhary Z., Subramaniam S., Khan G.M., Abeer M.M., Qu Z., Janjua T., Kumeria T., Batra J., Popat A. (2019). Encapsulation and controlled release of resveratrol within functionalized mesoporous silica nanoparticles for prostate cancer therapy. Front. Bioeng. Biotechnol..

[25] Ang C.W., Tan L., Qu Z., West N.P., Cooper M.A., Popat A., Blaskovich M.A. (2021). Mesoporous silica nanoparticles improve oral delivery of antitubercular bicyclic nitroimidazoles. ACS Biomater. Sci. Eng..

[26] Janjua T.I., Cao Y., Ahmed-Cox A., Raza A., Moniruzzaman M., Akhter D.T., Fletcher N.L., Kavallaris M., Thurecht K.J., Popat A. (2023). Efficient delivery of Temozolomide using ultrasmall large-pore silica nanoparticles for glioblastoma. J. Control. Release.

[27] Brown T.D., Habibi N., Wu D., Lahann J., Mitragotri S. (2020). Effect of nanoparticle composition, size, shape, and stiffness on penetration across the blood–brain barrier. ACS Biomater. Sci. Eng..

[28] Santra S., Yang H., Dutta D., Stanley J.T., Holloway P.H., Tan W., Moudgil B.M., Mericle R.A. (2004). TAT conjugated, FITC doped silica nanoparticles for bioimaging applications. Chem. Commun..

[29] Dong S., Roman M. (2007). Fluorescently labeled cellulose nanocrystals for bioimaging applications. J. Am. Chem. Soc..

[30] Souza T.G., Ciminelli V.S., Mohallem N.D.S. A comparison of TEM and DLS methods to characterize size distribution of ceramic nanoparticles. Proceedings of the Journal of Physics: Conference Series.

[31] Huang X., Shi X., Hansen M.E., Setiady I., Nemeth C.L., Celli A., Huang B., Mauro T., Koval M., Desai T.A. (2020). Nanotopography enhances dynamic remodeling of tight junction proteins through cytosolic liquid complexes. ACS Nano.

[32] Nugent M.A. (2022). The future of the COVID-19 pandemic: How good (or bad) can the SARS-CoV-2 spike protein get?. Cells.

[33] Peisahovics F., Rohaim M.A., Munir M. (2022). Structural topological analysis of spike proteins of SARS-CoV-2 variants of concern highlight distinctive amino acid substitution patterns. Eur. J. Cell Biol..

[34] Hashimoto Y., Campbell M. (2020). Tight junction modulation at the blood-brain barrier: Current and future perspectives. Biochim. Et Biophys. Acta (BBA)-Biomembr..

[35] Abbott N.J., Patabendige A.A., Dolman D.E., Yusof S.R., Begley D.J. (2010). Structure and function of the blood–brain barrier. Neurobiol. Dis..

[36] Finbloom J.A., Huynh C., Huang X., Desai T.A. (2023). Bioinspired nanotopographical design of drug delivery systems. Nat. Rev. Bioeng..

[37] Lockman P.R., Koziara J.M., Mumper R.J., Allen D.D. (2004). Nanoparticle surface charges alter blood–brain barrier integrity and permeability. J. Drug Target..

[38] Mo J., He L., Ma B., Chen T. (2016). Tailoring particle size of mesoporous silica nanosystem to antagonize glioblastoma and overcome blood–brain barrier. ACS Appl. Mater. Interfaces.

[39] Song Y., Du D., Li L., Xu J., Dutta P., Lin Y. (2017). In vitro study of receptor-mediated silica nanoparticles delivery across blood–brain barrier. ACS Appl. Mater. Interfaces.

