# Supplementary material for: Virus-like Silica Nanoparticles Improve Permeability of Macromolecules across the Blood–Brain Barrier In Vitro
Source: Pharmaceutics. 2023 Aug 30;15(9):2239. doi: 10.3390/pharmaceutics15092239 (PMC10536620; doi:10.3390/pharmaceutics15092239)
Supplement: Supplementary file 1 [file pharmaceutics-15-02239-s001.zip › pharmaceutics-2568718-supplementary.pdf]

Supporting Information

Virus-like Silica Nanoparticles Improve Permeability of Macromolecules across the Blood–Brain Barrier In Vitro

Yuran Feng, Yuxue Cao, Zhi Qu, Taskeen Iqbal Janjua * and Amirali Popat *

School of Pharmacy, The University of Queensland, Brisbane, QLD 4102, Australia
* Correspondence: t.janjua@uq.edu.au (T.I.J.); a.popat@uq.edu.au (A.P.)

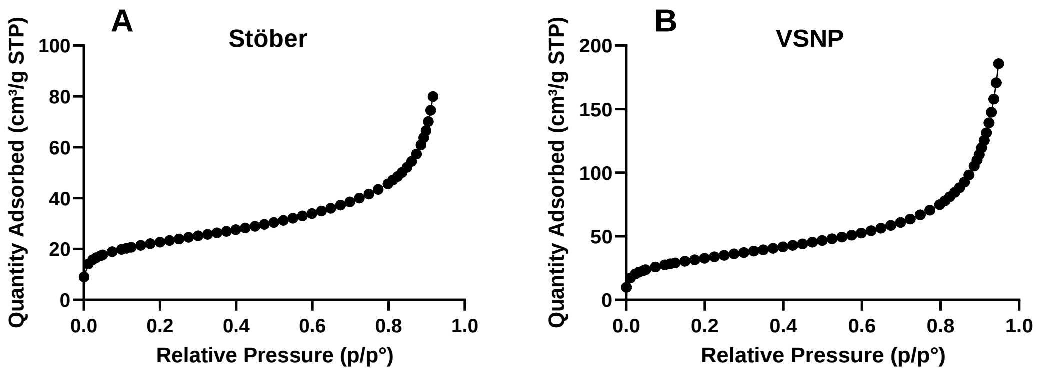

Figure S1. Nitrogen adsorption−desorption isotherms of (A) Stöber and (B) VSNP.

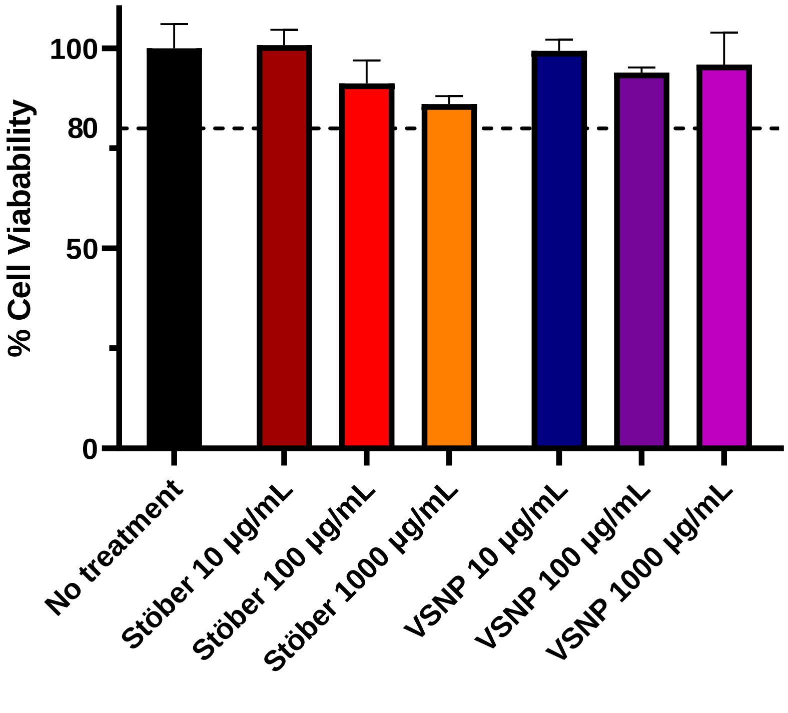

Figure S2. Cell viability was tested in hCMEC/D3 brain endothelial cell line. Cell treated with either Stöber and VSNP for 4 hours (concentration from 10µg/mL to 1000µg/mL) showed no significant toxicity and cell viability was above 80% at all concentrations. AlamarBlue assay was performed to study the cytotoxicity, n=5 for treatment groups and n=10 for no treatment group.

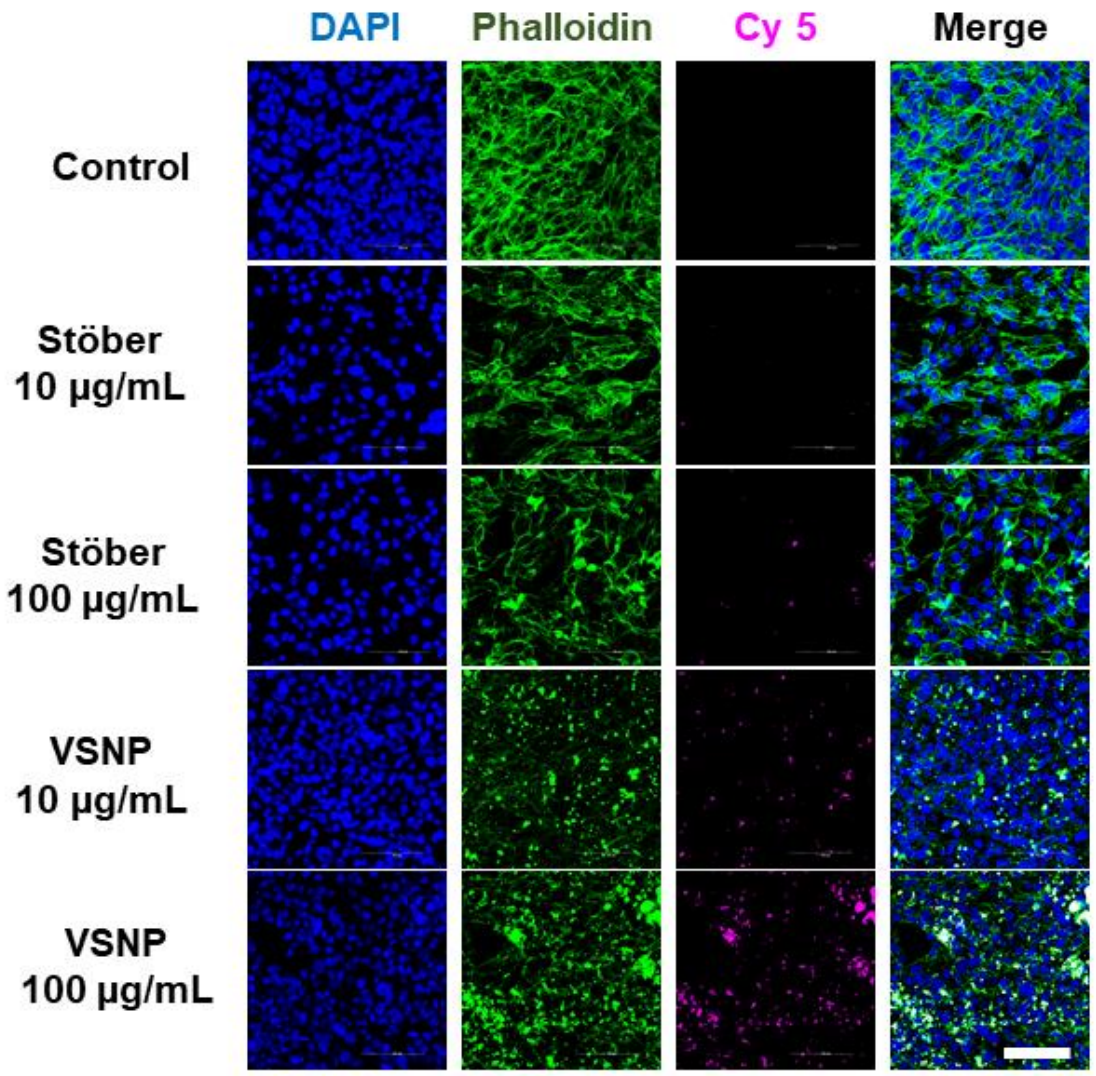

Figure S3. Uptake of Stöber and VSNP nanoparticles into human brain endothelial cells hCMEC/D3 analysed by confocal microscopy. Scale bar, 100 µm. Blue DAPI staining nuclei, green Phalloidin staining cytoskeleton and pink Cy5 labelled silica nanoparticles.
